# Bone marrow hemodilution assessment in multiple myeloma MRD by next generation flow cytometry - a mini review

**DOI:** 10.3389/fonc.2026.1849714

**Published:** 2026-05-01

**Authors:** Sorina Nicoleta Badelita, Horia Mihail Sandu, Daniel Coriu, Delia Codruta Popa

**Affiliations:** 1 Department of Hematology, Fundeni Clinical Institute, Bucharest, Romania; 2 Department of Hematology, “Carol Davila” University of Medicine and Pharmacy, Bucharest, Romania; 3 Department of Pediatrics, “Carol Davila” University of Medicine and Pharmacy, Bucharest, Romania

**Keywords:** blood, bone marrow, contamination, flow cytometry, hemodilution, MRD, multiple myeloma, review

## Abstract

Bone marrow hemodilution could compromise next generation flow cytometry based measurable residual disease evaluation in multiple myeloma and may cause false negative results. This review summarizes definitions, markers, indices, mitigation strategies, and current challenges in standardization.

## 1 Introduction

Multiple Myeloma (MM) is a hematological neoplasm characterized by an expansion of clonal plasma cells in the bone marrow (1, 2). It is the second most frequent hematological malignancy, representing approximately 1% of all cancers and approximately 10% of hematologic malignancies (3–5).

Measurable Residual Disease (MRD) in MM has major clinical relevance because MRD negativity is correlated with better Progression Free Survival (PFS) and Overall Survival (OS) (6), can guide treatment decisions and may also support treatment discontinuation in selected settings (7, 8). Next Generation Flow Cytometry (NGF) and Next Generation Sequencing (NGS) are the preferred methods for MRD detection. However, NGF offers broader applicability, lower cost, faster turnaround, and the intrinsic ability to assess hemodilution, whereas NGS requires fewer cells and is less time sensitive but is more expensive, less widely applicable, slower, and unable to evaluate hemodilution directly (9).

Dilution of bone marrow aspirate with peripheral blood (hemodilution) may decrease representation of bone marrow resident populations, including clonal plasma cells, and therefore MRD assessment could be distorted or even harbor false negative in heavily hemodiluted samples.

Even though the hemodilution is a widely recognized issue of MRD in MM, there is no standardized method of estimating, reporting or interpreting it. Multiple groups have proposed different adequacy markers or indices for hemodilution assessment.

## 2 Hemodilution definition and causes

Hemodilution is lowest in the first bone marrow pull, typically for the initial 2–4 mL, requires moderate aspiration and increases with sequential pulls or larger volumes (10, 11). Structural marrow changes that alter local blood flow, particularly fibrosis, further reduce true marrow yield and increase peripheral blood contamination (12).

Although hemodilution can affect MRD assessment in any hemato-oncologic disease, its impact is especially relevant in MM. This reflects several concurrent factors, including the patchy distribution of plasma cells in bone marrow, higher fragility compared to other cell subsets, and additional plasma cell loss during pre-analytical handling, which may together produce up to a 10 fold reduction or more in the plasma cell compartment (13, 14). Consequently, even modest relative increases in peripheral blood cell subsets due to hemodilution may cause false negative results in low burden MM MRD.

## 3 Cellular subpopulations used to assess bone marrow quality/hemodilution

Hemodilution in NGF is generally estimated using marrow associated populations, mainly mast cells (MC), nucleated red blood cells (NRBC), and B cell progenitors (BCP), sometimes together with composite indices, although this approach has not been applied uniformly across studies. Overall, MC appear to be the most practical single marker of hemodilution, but combined assessment with NRBC and BCP is likely more robust, particularly because treatment related depletion is a major confounder.

Puig et al. proposed one of the most comprehensive NGF based approaches by assigning reference values according to therapy, age, and disease status using MC, NRBC, and BCP (15). Differences related to patient age were small in healthy adults, supporting the general applicability of these populations outside major treatment related perturbations. By contrast, treatment had a substantial impact: BCP could be absent or markedly reduced after Rd or Dara-KRd, MC may be very low or absent after Rd, and simultaneous loss of MC, NRBC, and BCP is more frequently encountered after salvage CAR-T. These findings indicate that lymphodepleting or plasma cell specific therapies have the potential to markedly impair hemodilution assessment and increase the risk of falsely negative MRD interpretation when MRD is also undetectable (15). When prior therapy is unknown, age based healthy adult reference values could therefore serve as a practical comparator.

Soh et al. and Leonardos et al. likewise supported a multiparametric strategy, using MC, BCP, myeloid precursors, and NRBC for hemodilution assessment; Soh et al. concluded that at least two of these four populations should be available for reliable interpretation and proposed reference values for each subset, reinforcing the concept that marrow quality is better estimated through combined cellular parameters than by a single population alone (16, 17).

In contrast, de Pontes et al. used MC alone and defined hemodilution using a cutoff of ≤0.002%, showing that samples classified as hemodiluted had lower immature stage 1 BCP and lower normal plasma cells, whereas transitional and naive B cell subsets were not as affected (18). Foureau et al. also relied on MC as the sole marker and reported lower MC values during early treatment than at later time points, further emphasizing that therapy can impact the interpretation of this parameter (19). Together, these studies support MC as a practical warning marker, particularly around the recurrent <0.002% threshold, but also shows the limitations of using MC alone in different treatment scenarios.

More recent recommendations further support combined evaluation of MC, NRBC, and BCP, while suggesting that myeloid precursors should be interpreted cautiously because they may reflect myelodysplasia rather than hemodilution alone (20). Taken together, the literature suggests that MC are the most practical single surrogate marker for flow cytometric hemodilution assessment, but combined interpretation of MC, NRBC, and BCP looks more reliable, especially when therapy or disease characteristics may selectively deplete marrow specific populations (15, 18–20).

## 4 Hemodilution indices and composite scoring approaches

A comparative overview of the main hemodilution indices is presented in **Table 1**. Currently available approaches differ in logistical requirements, compatibility with standardized EuroFlow MM NGF workflows, and degree of validation in MM MRD (21). These methods can be divided in three groups: reference indices requiring paired peripheral blood and bone marrow data, flow cytometry based approaches that are not fully compatible with the standard EuroFlow MM MRD panel, and newer EuroFlow compatible indices that are still insufficiently validated in treated patients and MRD settings.

**Table 1:** Comparison of indices used for hemodilution assessment in different settings, their strenghts and their limitations.

Index name	Parameters used	Formula	Hemodilution groups	Paired PB required	Requires automated hematology counts	Compatible with EuroFlow MM NGF panel	MM MRD validated	Year	Reference method	Main limitation
Holdrinet Index (HI) (17)	Peripheral Blood and Bone Marrow Hemoglobin Value (HbPB & HbBM) & Peripheral Blood and Bone Marrow Nucleated Cell Counts (NCPB & NCBM)	HI = 100 - 100 * (HbBM/HbPB * NCPB/NCBM)	NA	YES	YES	NO	NO	1980	NO	requires paired PB and BM plus hematology analysis; not flow cytometry only
modified Holdrinet Index (mHI) (18)	Erythrocyte count in Peripheral Blood (RBCPB) and Bone Marrow (RBCBM) & White Blood Cell Count in Peripheral Blood (WBCPB) and Bone Marrow (WBCBM)	mHI = 100 * (1-RBCBM/RBCPB * WBCPB/WBCBM)	≥80% = good quality bone marrow; <50% = hemodiluted sample	YES	YES	NO	NO	2009	NO	same logistical limitation as HI; affected by altered erythropoiesis/myelopoiesis
IGRA/N ratio (22)	Immature Granulocytes (Promyelocytes, Myelocytes, Metamyelocytes) and Mature Neutrophils CD10+ CD16+	Immature Granulocytes/Mature Neutrophils	>1.2 = highly pure bone marrow; 0.5-1.2 = moderately pure bone marrow; <0.5 = low purity bone marrow	NO	NO	NO	NO	2018	NO	not EuroFlow NGF MM MRD panel compatible
ALLgorithMM (23)	IGRA/N & NRBC (5%) & Mast Cells (0.006%)	NO - see Figure 4 in the Original Article	Hemodiluted; Mildly Hemodiluted; Non-Hemodiluted	NO	NO	NO	YES	2022	NO	not Euroflow NGF MM MRD panel compatible; broader validation still needed
Bone Marrow Quality Index (BMQI) (28)	Myeloid Precursors (MP); Nucleated Red Blood Cells (NRBC)	BMQI = (% myeloid precursors × 0.37) + (% nucleated red blood cells × 0.14)	≥2.5, adequate quality; <2.5, hemodiluted; <1.4, considerably hemodiluted; and <0.5, severely hemodiluted	NO	NO	YES	NO	2023	NO	validated only in untreated diagnostic MM

* = mathematical function of multiplication.

The earliest reference methods were the Holdrinet Index (HI) and its later modification, the modified Holdrinet Index (mHI). Holdrinet et al. first demonstrated that a substantial proportion of hemoglobin in bone marrow aspirates derives from peripheral blood contamination and developed an index based on paired peripheral blood and bone marrow hemoglobin and nucleated cell counts (22). Brooimans et al. later adapted this concept by replacing hemoglobin and nucleated cell counts with erythrocyte and white blood cell counts, effectively creating the mHI (23). These methods remain important reference frameworks, but the routine use in MM MRD is cumbersome because they require paired peripheral blood sampling and automated hematology analysis of both marrow and blood, making them poorly suited to standardized NGF workflows in a normal hematology flow cytometry department.

These logistical limitations are particularly relevant in MM, where marrow compartment may be substantially altered by disease and treatment. Although mHI was initially validated only in normal marrow, later work supported its use in myeloid neoplasms for CD34 positive cell normalization (24). However, neither HI nor mHI directly addresses treatment related perturbations of erythropoiesis or myelopoiesis, which are frequent in MM at diagnosis and across treatment specific stages (25, 26). Thus, while HI and mHI are useful as reference standards, the applicability in routine MM NGF MRD is limited.

A second group of papers includes flow cytometry based indices that do not use paired peripheral blood but are not fully compatible with the standardized EuroFlow MM MRD panel. The IGRA/N ratio, introduced by Pont et al., was developed to distinguish non-hemodiluted from hemodiluted marrow using the ratio of immature granulocytes to mature neutrophils and was validated against mHI (27). Building on this framework, Vigliotta et al. proposed ALLgorithMM, which refined IGRA/N by incorporating MC and NRBC to improve classification of hemodilution in MM MRD and other lymphoproliferative diseases (28). These approaches are attractive because they are flow cytometry based and do not require paired peripheral blood; the main limitation is the fact that they depend on markers outside the standard EuroFlow MM MRD assay, which is a limiting factor for the streamlined implementation in standardized routine practice.

Similar limitations apply to the Peripheral Blood Contamination Index (PBCI), developed by Delgado et al., which incorporates CD34 positive cells, normal plasma cells, and CD10 positive neutrophils (29). Although the index was designed so that two preserved populations might still support assessment when one is altered, this assumption is problematic in MM MRD. Normal plasma cells are not reliable adequacy markers in this setting, and newer therapies may simultaneously affect more than one component of the index, particularly with anti-CD38 antibodies exposure that may also target CD38 positive BCP (30). Concurrent myelodysplastic syndrome may further distort CD34 positive and CD10 positive compartments (31, 32). Accordingly, PBCI is of conceptual interest but has limited practical value in contemporary MM NGF MRD workflows.

The most promising EuroFlow compatible approach to date is the Bone Marrow Quality Index (BMQI), introduced for newly diagnosed MM; it is based on NRBC and myeloid precursors measured in first and second pull aspirates (33). NRBC emerged as the strongest discriminator of marrow quality, followed by myeloid precursors, so both populations were incorporated into the score. The main strength of BMQI is its compatibility with the EuroFlow MM NGF panel. The main limitation is the fact that its validation was restricted to untreated diagnostic samples, therefore its performance in post treatment MRD settings is uncertain because therapy can selectively alter the same cellular compartments used for the index calculation.

Currently available indices should be interpreted according to their intended role rather than viewed as interchangeable tools. HI and mHI remain useful reference standards but are logistically cumbersome. IGRA/N, ALLgorithMM, and PBCI are flow cytometry based and avoid paired peripheral blood, but their dependence on non EuroFlow markers limits routine applicability. BMQI is the most attractive EuroFlow compatible candidate, yet it still lacks validation in treated MM MRD cohorts. At present, no single index can be considered sufficient as a standalone standard for hemodilution assessment in NGF based MM MRD. Instead, composite indices should be regarded as complementary tools, and their interpretation must remain integrated with other specimen quality markers, treatment context and marrow specific cellular populations.

## 5 Strategies to mitigate hemodilution in multiple myeloma MRD testing

EuroFlow NGF requires acquisition of more than 10 million events to achieve 10^-5^ sensitivity, so the full processing of the first bone marrow aspirate becomes essential. Bulk lysis remains the standard pre-analytical approach because it has multicentric validation in normal, pathological and treatment related settings, removes most red blood cells and concentrates white blood cells to maximize cell recovery. In MM it is clinically important because improved specimen quality reduces hemodilution related false negative MRD results. Even though other alternative strategies have been proposed to enhance plasma cell recovery or reduce dilution effects, none has yet replaced bulk lysis in routine practice.

Density gradient separation represents the best studied alternative enrichment strategy. Compared with bulk lysis or red blood cell lysis, it efficiently eliminates red blood cells and granulocytes and could improve plasma cell recovery (14, 34). O’Brien et al. also explored negative selection to deplete common cell populations while preserving BCP and plasma cells, but this approach increases costs and processing time and may also remove CD33 positive myeloma cells from further analysis (35). Thus, while density gradient based enrichment is promising, it remains insufficiently validated to replace the current standard.

A different strategy is positive selection with anti-CD38 and/or anti-CD138 magnetic beads, as investigated in protocols like the BloodFlow protocol, which aims to recover circulating plasma cells from peripheral blood rather than bone marrow (36). This approach is conceptually attractive because it may achieve 10^-7^–10^-8^ sensitivity in peripheral blood. However, positive selection is generally approached with caution because it may alter immunophenotype, and its role remains investigational rather than practice changing, at least as of now.

Other methods aim to improve cell recovery without relying on bulk lysis. The dextran sedimentation method induces spontaneous red blood cell aggregation while preserving other cellular populations, thereby concentrating the sample without RBC lysis (37). The pooled-tube method divides whole bone marrow into several tubes for washing and staining before recombination into a single acquisition tube. Both methods have been reported to reach the International Myeloma Working Group (IMWG) recommended the minimum acquisition of 5 million cells, but their practical value in standardized MM MRD workflows remains uncertain (38).

An additional concept is to interrogate the particle or spicule fraction of bone marrow aspirates, which is usually discarded during pre-analytical processing despite the fact that it could containing high numbers of normal and clonal plasma cells. Zhang et al. developed a method for particle enrichment, disaggregation, and flow cytometric analysis, and Jiang et al. subsequently validated this strategy for MRD assessment in MM (39, 40). However, the reported sensitivity was 10^-4^, below the recommended 10^-5^ threshold, which currently limits its applicability.

Overall, the available alternatives to bulk lysis differ in purpose: some improve enrichment of the liquid fraction, some target otherwise discarded marrow compartments, and others attempt to shift plasma cell detection to peripheral blood. Despite these innovations, bulk lysis remains the practical standard for NGF based MM MRD because it combines standardization, multicenter validation, and compatibility with current workflows. Alternative approaches are promising, but they are not yet sufficiently validated to replace present practice.

## 6 Discussion

Multiple myeloma is a leading model for MRD based response assessment, with NGF and NGS representing the principal high sensitivity bone marrow methods, complemented by imaging and mass spectrometry for disease evaluation within and beyond the marrow compartment (41). Analytical sensitivity alone does not ensure clinical reliability. False negative results remain a major limitation of bone marrow based MRD analysis; PET-CT may identify disease missed by NGF or NGS, underscoring the impact of sampling bias and different affected sites heterogeneity (15). Within this context, hemodilution is a critical pre-analytical variable because it can reduce the representation of clonal plasma cells in bone marrow aspirates and also compromise detection of clonal rearrangements by NGS (42). Accordingly, the ability of NGF to assess both MRD and specimen quality represents a major practical advantage over NGS, which cannot directly evaluate hemodilution, but the cell requirement for reliable analysis is tens of times higher in NGF when compared to NGS (9).

Routine hemodilution assessment is mandatory in NGF based MM MRD, but no international standard exists (15). Current evidence favors combined marrow specific cells like MC, NRBC, BCP, and myeloid precursors over binary or single marker approaches; MC alone, when using the recurrent <0.002% cut-off value, are useful mainly as a warning hemodilution signal, but should not be used alone (15, 16, 18–20). Interpretation must take into account the treatment regimen, since Rd, Dara-KRd, and salvage CAR-T could markedly deplete the populations used for hemodilution estimation and increase the risk of false negative MRD (15).

Plasma cells as adequacy markers are even less reliable for hemodilution assessment in MM MRD context. Low plasma cell counts may reflect treatment rather than hemodilution, and the pathological/total plasma cell ratio is largely restricted to diagnosis and could be severely influenced by pull order, CD56 related adhesion effects and other pre-analytical factors (13, 14, 16, 19, 20). Composite indices are more formalized but still limited: HI and mHI require paired peripheral blood and hematology analyzer count; IGRA/N and ALLgorithMM need non-EuroFlow markers; BMQI, even though EuroFlow compatible, it remains validated only for untreated diagnostic samples (22, 23, 27, 28, 33).

Practically, evidence supports a hierarchical interpretation strategy. First, MRD results should always be interpreted in light of specimen adequacy. Second, combined assessment of MC, NRBC, and BCP currently appears more robust than reliance on any single population. Third, therapy related depletion of marrow specific cells should be explicitly considered before labeling a specimen as hemodiluted or adequate. Finally, when possible, composite indices may complement but should not replace biologically informed interpretation, particularly in post treatment MRD samples where marrow reconstitution patterns may be highly atypical (15, 16, 20, 33).

Reporting practices should also be standardized. In line with IMWG recommendations, the first bone marrow aspirate should be prioritized for NGF, sensitivity should exceed 10^-5^ with acquisition of at least 5 million viable cells, and Limit of Detection (LOD) and Lower Limit of Quantification (LLOQ) should be reported as percentages (for 30 and 50 events cut-off values) (21, 38). Hemodilution status should be incorporated into MRD reports whenever possible, because an undetectable MRD result in a severely or moderately hemodiluted bone marrow sample should not carry the same interpretive weight as an undetectable MRD result in a high quality bone marrow sample (15). Consistent with current terminology recommendations, “undetectable MRD” is preferable to “negative MRD” in the patient’s results report (41).

Overall, the field is moving toward more integrated assessment of residual disease in MM, combining bone marrow based MRD with peripheral blood analysis and imaging, proteomic and genetic approaches (15, 41). In that setting, hemodilution assessment will become even more important because bone marrow results will increasingly need to be interpreted against other disease compartments. Future work should therefore prioritize harmonized reporting criteria, validation of EuroFlow compatible hemodilution tools in treated MRD cohorts (including automated analysis), and development of standardized interpretive frameworks that integrate treatment context, sample quality, and MRD status (21). Automated models may contribute to this effort, but at present they remain exploratory in MM (43).
